# PATENT DUCTUS ARTERIOSUS CLOSURE: EXPERIENCE FROM A TERTIARY REFERRAL CENTER

**DOI:** 10.1590/1984-0462/2021/39/2020013

**Published:** 2020-11-27

**Authors:** Diogo Remi Oliveira Faim, Joaquim António Maia Tiago, Rui Jorge Simões Castelo, Andreia Sofia Santos Francisco, Rosa Ramalho Alves, António Manuel Guerra Santos Pires

**Affiliations:** a Universitário de Coimbra, Coimbra, Portugal.

**Keywords:** Ductus arteriosus, patent, Infant, premature, Ibuprofen, Acetaminophen, Neonatology, Canal arterial persistente, Recém-nascido prematuro, Ibuprofeno, Acetaminofen, Neonatologia

## Abstract

**Objective:**

To characterize the number and methods of closure of Persistent Ductus Arteriosus (PDA) over a span of 16 years in a third level maternity hospital.

**Methods:**

Retrospective study of neonates born between January 2003 and Deccember 2018, who underwent ductus arteriosus closure by pharmacological, surgical and/or transcatheter methods. Gestational age, birth weight, number and methods of closures per year were evaluated. The success rate of the pharmacologic method was calculated, as well as the mortality rate. The association between mortality and birthweight, treatment used and treatment failure was explored.

**Results:**

There were 47,198 births, 5,156 were preterm, 325 presented PDA and 106 were eligible for closure (median gestational age - 27 weeks, birthweight <1000 g - 61%). Frequency of PDA closure decreased during the study period, especially starting in 2010. Success rate with pharmacologic treatment was 62% after the first cycle and 74% after the second. After drug failure, 12 underwent surgical ligation and two underwent transcatheter closure. Exclusive surgical ligation was indicated in four infants. Ibuprofen replaced indomethacin in 2010, and acetaminophen was used in three infants. Among the 106 infants, hospital mortality was 12% and it was associated with birthweight <1000 g (13/65 <1000 vs. 0/41 >1000 g; p=0.002) and with failure in the first pharmacologic treatment cycle (13/27 with failure, vs. 0/75 without failure; p<0.001).

**Conclusions:**

The national consensus published in 2010 for the diagnosis and treatment of PDA in preterm infants led to a decrease in the indication for closure. Pharmacological closure was the method of choice, followed by surgical ligation. Birthweight <1000 g and first cycle of pharmacologic treatment failure were associated with higher mortality.

## RESUMO

**Objetivo:**

Caraterizar o número e métodos de fechamento de canal arterial durante 16 anos numa maternidade de nível terciário.

**Métodos:**

Estudo retrospetivo de nascidos entre 01 de janeiro de 2003 a 31 de dezembro de 2018 submetidos a fechamento do canal arterial por métodos farmacológico, cirúrgico e/ou percutâneo. Avaliaram-se idade gestacional, sexo, peso ao nascimento, número de fechamentos por ano e método utilizado. Aferiram-se as taxas de sucesso de método farmacológico e de mortalidade e sua associação com peso ao nascer, fármaco utilizado e insucesso do fechamento.

**Resultados:**

Verificaram-se 47.198 recém-nascidos, 5.156 prematuros, dos quais 325 com canal arterial patente, sendo 106 com indicação para fechamento (idade gestacional mediana 27 semanas, peso <1000 g em 61%). Verificou-se diminuição do número de fechamentos ao longo dos anos, sobretudo a partir de 2010. O fechamento ocorreu em 62% após primeiro ciclo de tratamento farmacológico e em 74% após segundo. Após insucesso farmacológico, 12 realizaram ligadura cirúrgica e dois, fechamento percutâneo. Houve indicação de ligadura cirúrgica exclusiva em quatro. O ibuprofeno substituiu a indometacina em 2010. O acetaminofen foi usado em três doentes. A mortalidade nos 106 pacientes foi de 12%, associando-se ao peso ao nascer (13/65 <1000 vs. 0/41 >1000 g; p=0,002) e à falha do primeiro ciclo de tratamento farmacológico (13/27 com falha vs. 0/75 com sucesso; p<0,001).

**Conclusões:**

Consenso nacional de 2010 para diagnóstico e tratamento do canal arterial nos prematuros levou à diminuição do número de fechamentos desse canal. O fechamento farmacológico foi o método mais utilizado, seguido da ligadura cirúrgica. Peso <1000 g e falha no primeiro ciclo de fechamento farmacológico se associaram à maior mortalidade.

## INTRODUCTION

The ductus arteriosus (DA) allows for communication between the aorta and the pulmonary arteries. During the fetal period, it permits oxygenated blood that comes from the maternal placenta to circulate around the system, as it is conditioned by the high pulmonary vascular resistance that exists during this period.^1^ After birth, the increase in partial arterial oxygen pressure (PaO2) and the decrease of circulating prostaglandins (PG) induce a constriction of the middle wall of the DA.^2^ In term newborns (NB), the DA usually occludes up to 48 hours after birth. Failure for the DA to close at 72 hours of life is called patent ductus arteriosus (PDA).^3^ In premature newborns, the prevalence of PDA is higher and, when hemodynamically significant, it is associated with higher rates of mortality and comorbidities, especially pulmonary hemorrhage, bronchopulmonary dysplasia, intraventricular hemorrhage and necrotizing enterocolitis, when compared to premature infants without PDA or with non-hemodynamically significant PDA. On the seventh day of life, 87% of newborns at 24 weeks of gestational age (GA) and 64% of newborns at 27 to 28 weeks of GA continue to have their canal open.^5^ There are some documented risk factors for PDA, such as excessive intake of intravenous fluids in the first week of life, prolonged rupture of membranes, sepsis and administration of furosemide.^1^ The approach and treatment of PDA have evolved in recent years using pharmacological,^6^ ^,^ ^7^ ^,^ ^8^ surgical^9^ and transcatheter closure methods.^10^ ^,^ ^11^ ^,^ ^12^

The primary objective of this study was to evaluate the modification of the number and methods of DA closure in premature newborns over the years, at the Differential Perinatal Support Unit (*Unidade de Apoio Perinatal Diferenciado* - UAPD) of a level three maternity hospital, over a period of 16 years. As secondary objectives, with this sample of premature infants, it was possible to measure the success rates of closure with indomethacin and ibuprofen and assess associations with hospital mortality.

## METHOD

All premature newborns born in a maternity ward at the UAPD between January 1, 2003 and December 31, 2018 were included. Such a unit is able to receive and treat all at-risk NBs, regardless of GA, as well as perform all treatments and procedures, with the exception of neonatal surgery and subspecialties involving advanced technology. Of those born in the unit, those without PDA were excluded. Among those who exhibited this condition, those who did not have a hemodynamically a significant ductus arteriosus canal, as well as those who had other concomitant congenital heart diseases or associated genetic syndromes, were eliminated. All patients with hemodynamically significant PDA (PDA-HS) underwent an attempt to close the canal using a pharmacological method, a surgical ligation method, or a transcatheter method. Surgical ligations designed to close the DA were performed at the Pediatric Intensive Care Unit (PICU) in the same hospital. These NBs were also included in the study.

The diagnosis of PDA-HS was made by the pediatric cardiologist, using a transthoracic echocardiogram. Before 2010, this diagnosis was based on the existence of PDA and subjective evidence of dilation of the left heart cavities on a transthoracic echocardiogram. After 2010, national consensus criteria were put into place to considered the diagnostic and therapeutic approach of PDA in preterm infants,^13^ namely: a minimum diameter of the DA> 1.4 mm/kg of weight, non-restrictive left-right flow in the DA, left atrium/aorta ratio> 1.4, absent or retrograde diastolic flow in the descending aorta.

Pharmacological closures, surgical ligation closures, and transcatheter closures were included. Pharmacologists used indomethacin, ibuprofen or acetaminophen. The amount of indomethacin used was three doses of 0.2 mg/kg/intravenous dose (IV) every 12 hours. For ibuprofen, a dose of 10 ­mg/­kg IV followed by two doses of 5 mg/kg IV at 24h intervals. For acetaminophen, the regimen applied was 15 mg/kg/dose every six hours for three days. Surgical ligation was suggested when there was no response to pharmacological treatment. It was the first option considered when other concurrent surgeries were needed or when there were contraindications for pharmacological treatment, such as oliguria, acute renal failure, thrombocytopenia, active hemorrhaging or necrotizing enterocolitis. Transcatheter closure was considered when there was no response to pharmacological treatment, surgical contraindication, and weight was greater than 1 kg. NBs with non-hemodynamically significant PDA were excluded from this study, and were treated with a conservative approach, such as using water restriction.

All NBs were evaluated by the pediatric cardiologist using transthoracic echocardiograms at the end of the selected treatment. After a pharmacological treatment attempt was made, the newborns who still had PDA-HS criteria underwent a new attempt at closure, according to indications and a multidisciplinary medical decision. NBs who maintained non-hemodynamically significant PDA received monitoring and fluid restriction.

To characterize the population, data such as gestational age, sex, birth weight and mortality were collected. The success rates of pharmacological closure were calculated after each cycle of pharmacological treatment, separately, for indomethacin and ibuprofen. Associations between mortality and birth weight greater or less than 1000g, between mortality and type of drug used and between mortality and closure from surgical ligation were investigated.

Statistical analysis was performed using the Statistical Package for the Social Sciences (SPSS) program (IBM^®^, SPSS^®^ Statistics Inc., Chicago), version 25.0. The Shapiro-Wilk test was used to test normality. Continuous variables with normal distribution were described by means and standard deviation and continuous variables without normal distribution were described using median and interquartile range (IQR). In order to verify the associations between mortality and the various variables, the Chi-square and Fisher’s tests were used to compare nominal variables. Results were considered significant if p<0.05.

## RESULTS

During the study period, 47,198 newborns were born in the maternity hospital, of which 5,156 (11%) were premature. The relationship between the number of total births and premature newborns in this maternity hospital, per year, is shown in Graph 1. A total of 4,341 newborns were admitted to the UAPD of the maternity hospital, and, among them, 2,767 (64%) were premature infants. A total of 325 NB with PDA were diagnosed at the PICU, of which 106 (33%) were considered hemodynamically significant, with indication for closure. In Graph 2, the number of NB who underwent DA closure per years, regardless of the method is represented. Of the NBs with treated DA, 52% were male. All newborns were premature, with a median GA of 27 weeks (IQR 26:29). Table 1 shows patients grouped into gestational age ranges. NBs with less than 28 weeks of GA and treated DA accounted for 24% of NB admitted to the PICU with this GA. Of the NB with a GA between 28 and 32 weeks, 4% tried to close the DA, while in the group with 33 to 37 weeks of GA, only 0.1% tried to close the DA. The median birth weight was 940g (IQR 750: 1500) and 61% weighed less than 1000 g.

**Figure 1:**
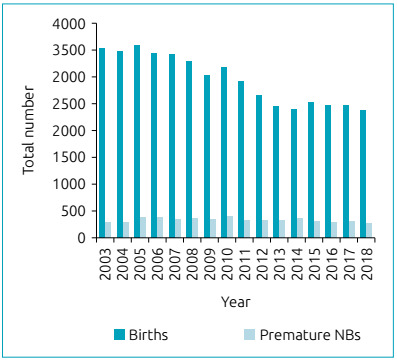
Total number of births and premature newborns, per year, from 2003 to 2018, in a maternity hospital.

**Figure 2:**
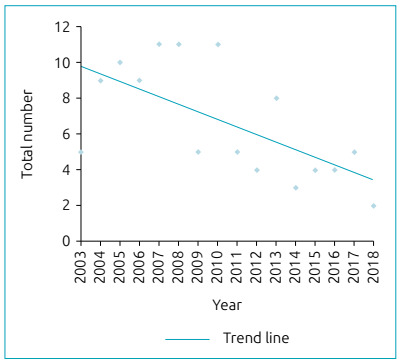
Number of newborns with pharmacological and/or surgical and/or transcatheter closure of the ductus arteriosus per year, from 2003 to 2018.

**Table 1:** Relationship between the total number of newborns admitted to the Differentiated Perinatal Support Unit and the number of newborns with hemodynamically significant ductus arteriosus, by gestational age.

Gestational age (weeks)	Total (n)	PDA closure [n (relative%)]
<28	249	61 (24)
[28-32]	1,077	43 (4)
[33-36]	1,441	2 (0.1)

n: absolute value; PDA: patent ductus arteriosus.

Of the 106 NBs with treated DA, 88 underwent an exclusively pharmacological approach, 12 underwent surgical ligation after the initial pharmacological approach, 4 underwent an exclusively surgical ligation approach, and in two, the closure was transcatheter after attempted pharmacological closure. Table 2 shows the closure methods performed, per year. Among the 102 NBs in which pharmacological treatment was attempted, indomethacin was used in 62, ibuprofen in 38 and acetaminophen in 3 (one patient had a first cycle with acetaminophen and a second cycle with ibuprofen). Table 3 illustrates the use of each drug over the years.

**Table 2:** Number of newborns who underwent closure of the ductus arteriosus, by year and by type of closure.

Year	Only pharmacological	Pharmacological and surgical	Pharmacological and transcatheter	Only surgical	Total
2003	3	2	0	0	5
2004	8	1	0	0	9
2005	10	0	0	0	10
2006	8	1	0	0	9
2007	10	1	0	0	11
2008	10	1	0	0	11
2009	3	2	0	0	5
2010	9	2	0	0	11
2011	4	0	1	0	5
2012	1	1	1	1	4
2013	7	0	0	1	8
2014	2	0	0	1	3
2015	2	1	0	1	4
2016	4	0	0	0	4
2017	5	0	0	0	5
2018	2	0	0	0	2
Total	88 (83%)	12 (11%)	2 (2%)	4 (4%)	106 (100%)

**Table 3:** Drugs used to close the ductus arteriosus, per year.

Year	Indomethacin	Ibuprofen	Paracetamol	Total
2003	5	0	0	5
2004	9	0	0	9
2005	10	0	0	10
2006	9	0	0	9
2007	11	0	0	11
2008	11	0	0	11
2009	5	0	0	5
2010	2	9	0	11
2011	0	5	0	5
2012	0	3	0	3
2013	0	7	0	7
2014	0	2	0	2
2015	0	3	0	3
2016	0	4	0	4
2017	0	4	2	6
2018	0	1	1	2
Total	62 (61%)	38 (37%)	3 (3%)	103 (100%)

The closure rate after the first cycle of pharmacological therapy was 62%. Of the remaining 38, 26% died before attempting a new type of closure, 66% repeated a second cycle and 8% proceeded to surgical ligation. The closure rate after the second pharmacological cycle was 48%. The closure rates after the first cycle with indomethacin and ibuprofen were 67 and 59%, respectively. In total, 74% of the patients managed to have their canal closed after one or two cycles of exclusively pharmacological therapy. Of the three patients for whom acetaminophen treatment was performed, one closed after two cycles, one’s DA remained open at the end of the first cycle and underwent a second cycle with ibuprofen, and the third died after one cycle.

Death occurred in 13 (12%) newborns, 11 of them in the neonatal period. The median day of death was 12 (IQR 6:19). There was an association between mortality and birth weight less than 1000 g (13/65 <1000 vs. 0/41 1000 g; p = 0.002) and between mortality and failure of the first pharmacological closure cycle (13/39 with failure of the first pharmacological closure cycle, vs. 0/63 with success of the first pharmacological closure cycle; p <0.001). There was no relationship between mortality and use of indomethacin (9/62 with indomethacin vs. 4/40 without indomethacin; p=0.504), ibuprofen (3/38 with ibuprofen vs. 10/64 without ibuprofen; p=0.362), paracetamol (1/3 with paracetamol vs. 12/99 without paracetamol; p=0.339) or surgical connection (0/16 with a surgical ligation vs. 13/90 without a surgical ligation; p=0.139). Among the causes of neonatal death, five were counted as having a cerebral intraventricular hemorrhage, three as having a pulmonary hemorrhage and three as having sepsis and multiple organ failure.

## DISCUSSION

The treatment of PDA in premature NBs has been the subject of debate and controversy for many decades.^14^ On the one hand, PDA-HS has been associated with serious comorbidities,^3^ ^,^ ^4^ however, closure methods, regardless of the type of method, also come with risks.^12^ ^,^ ^15^ ^,^ ^16^ There is not always a consensus as to what gives the best prognosis for newborns, whether to wait or to close the DA. Sathanandam et al. ^17^, through a survey study, concluded that pediatric cardiologists are more in favor of the benefits of closure of the DA than neonatologists. With greater knowledge of the pathophysiology of DAs, more conservative approaches have emerged.^18^ If no attempt is made to close the DA, the average day it closes on its own, in an RN with less than 26 weeks of GA, is 71 days, and this decreases to 13 days if the GA is between 26 and 28 weeks old.^19^ Over the 16 years of the study, 106 patients were considered to have PDA-HS, and closure was indicated. However, 67% of the closures occurred in the first eight years of life. As shown in Graph 2, there has been a decrease in the number of DA closures over the years. This trend is not only explained by the decrease in the number of births, as illustrated in Graph 1, since the number of premature newborns has remained stable, which is also shown in the graph. Since PDA-HS affects premature NBs, this implies that the trend is due to medical conduct and not to reduced birth rates. An approach that is more conservative has been verified essentially from 2010, that is, in the second half of this study. In 2010, the national consensus was approved for the diagnostic and therapeutic approach of PDA in preterm infants,^13^ which, in 2012, became a standard of the Directorate-General for Health (*Direção-Geral de Saúde* - DGS).^20^ The fact that only 33% of DAs were treated after 2010 shows that these guidelines had a direct impact on medical decisions. The requirement of diagnostic criteria and precise indications for therapy led to the lowest number of diagnoses of PDA-HS, resulting in a lower number of ductus arteriosus treated.

As shown in Table 2, pharmacological closure was the first choice in most cases. In four cases, surgical ligation was used from the start because there was a contraindication to ibuprofen therapy, such as thrombocytopenia (n = 2), or because the patients were subjected to another concomitant surgery (n=2). Avoiding surgery as the first option is based on the various complications described, such as vocal cord paralysis and postoperative hypotension,^1^ diaphragm paralysis,^21^ neurodevelopment disorder,^22^ pneumomediastinum, and chylothorax.^9^

Although pharmacological therapy appears to be less harmless, is not without risks. As a result, efforts have been made to find a drug that, in addition to being effective, is safe. The results presented in Table 3 reveal that indomethacin was the drug of choice until 2010. Starting that year, and coinciding with the publication of the national consensus, ibuprofen replaced it. Despite the similar efficacy between these two medications^6^ indomethacin has been associated with a higher incidence of serious adverse reactions, such as acute and chronic renal failure,^23^ hyperkalaemia, brain damage and enterocolitis necrotizing.^24^ For six years ibuprofen was used exclusively. In 2017, there was a need for analgesia in newborns with PDA-HS, and as such, the first use of acetaminophen in this pathology appeared. It was considered to be beneficial to use only one drug for both purposes. Several studies have shown that this drug has an efficacy similar to that of ibuprofen,^6^ ^,^ ^7^ ^,^ ^15^ ^,^ ^25^ ^,^ ^26^ ^,^ ^27^ but with a lower incidence of acute renal failure, oliguria,^15^ platelet dysfunction,^6^ gastrointestinal bleeding, hyperbilirubinemia^27^ and necrotizing enterocolitis.^.^ ^7^ Oncel et al.^8^ reported that there are still no differences in neurological prognosis at 18 and 24 months. In that study, this was the only drug used in three patients, and it was too early to draw conclusions. The success rate after pharmacological therapy was 62% after the first therapeutic cycle, rising to 74% after the second. The results after the first cycle fall short of that described in the literature.^1^ ^,^ ^7^ A possible explanation is the fact that, in this study, the reopening of the ductus arteriosus after pharmacological closure was considered to be a failure of the initial treatment and not as a recurrence after a successful closure. Although the closure rate with indomethacin (67%) was slightly higher than that with ibuprofen (59%), this difference was not significant, since more patients used indomethacin than ibuprofen.

A third possible approach, which circumvents adverse drug reactions and surgical complications, is transcatheter closure of the DA. It is a closure method that is already widely used in older children, even considered as a first line above 6 kg.^12^ In 2011, Bentham et al.^10^ reported a new echo-guided closure technique in newborns with very low birth weight. As a result, this closure method has become an increasingly valid option for premature NBs, with a reported success rates of over 80%.^11^ ^,^ ^28^ Despite possible complications, such as device embolization, cardiac tamponade and iatrogenic aortic coarctation,^9^ newborns with transcatheter closure have less cardiac improvement and more cardiac impairment of respiratory functions, compared to newborns with surgical closure.^16^ In the UAPD in question, the transcatheter method was used to close the DA in two newborns, both after two cycles with ibuprofen an no success. The device that was successfully used was the Amplatzer Duct Occluder II AS.

Mortality was 13 (12%) newborns, and 11 (85%) newborns in the neonatal period. These deaths occurred exclusively in patients weighing less than 1000 g at birth (13/65 <1000 vs. 0/41> 1000 g; p=0.002) and exclusively in patients in whom the first DA closure cycle failed (13/39 with failure of the first pharmacological closure cycle vs. 0/63 with success of the first pharmacological closure cycle, p<0.001). The fact that the success rate improved from 62 to 74% from the first to the second cycle shows that it was worth trying a second cycle. However, not having closed on the first attempt seems to be an indicator of a poor prognosis. Among neonatal deaths, eight occurred after pathologies associated with PDA-HS in premature newborns: cerebral hemorrhage (n = 5) and pulmonary hemorrhage (n = 3). Despite being pathologies with a higher prevalence in patients with PDA-HS, they are exclusively associated with PDA-HS at these gestational ages. It is important to note that patients with extremely low birth weight have many comorbidities^29^ and higher mortality rates^30^ than those of more mature newborns. In these patients, the existence of PDA-HS is another contributor to their instability, as it is difficult to attribute cause of death exclusively to one factor. No necropsy was asked for by the parents in any of the cases; consequently, PDA-HS cannot be considered as the exclusive cause of death in these patients, nor death as an adverse reaction to treatment for closure.

The limitations of the study are inherent to the fact that it is retrospective. In a six-year survey, patients may have been omitted due to coding errors or loss of information. The possible transfer of patients to other hospital centers, for various reasons, could have also led these patients to be counted in the births of the maternity hospital, but not in the diagnosis of PDA-HS. The subjectivity of the diagnosis of PDA-HS before 2010 could be an important source of bias. Furthermore, the diagnosis was not always made by the same pediatric cardiologist. Lastly, the ultrasound images used at the beginning of the study were not necessarily the same quality as those used in more recent years. Finally, although pharmacological closures have been second in number according to methods with the greatest consensus in the literature, there are other methods reported, which could have given other results.

Despite the limitations, the present study demonstrates the importance and the impact of a national guideline showing how to approach ductus arteriosus in newborns, allowing for less invasive procedures in a high-risk population, by clarifying diagnostic and therapeutic criteria. There is a trend towards a decrease in ductus arteriosus closures in premature newborns. This reduction stands out as starting in 2010, which coincided with the publication of the national consensus that was then later converted into a DGS standard. The drug used most often was indomethacin, but it was replaced in the same year by ibuprofen, which is just as efficient. The infrequent use of acetaminophen still does not allow for conclusions to be drawn about this drug, but globally the studies are promising. Although there is still a preference for surgical ligation when there are contraindications or failures in pharmacological treatment, first steps have been successfully taken with regard to the transcatheter approach to DA in newborns with very low birth weight. PDA is yet another comorbidity among many that patients with low birth weight are subject to, and it requires a close relationship between pediatric cardiology and neonatology.
